# Identification of metastasis-related genes for predicting prostate cancer diagnosis, metastasis and immunotherapy drug candidates using machine learning approaches

**DOI:** 10.1186/s13062-024-00494-x

**Published:** 2024-06-25

**Authors:** YaXuan Wang, Bo Ji, Lu Zhang, Jinfeng Wang, JiaXin He, BeiChen Ding, MingHua Ren

**Affiliations:** Department of Urology, The First Affiliated Hospital of Harbin Medical University, Harbin, 150001 China

**Keywords:** Prostate cancer, Metastasis, Machine learning, Prognosis, POLD1

## Abstract

**Background:**

Prostate cancer (PCa) is the second leading cause of tumor-related mortality in men. Metastasis from advanced tumors is the primary cause of death among patients. Identifying novel and effective biomarkers is essential for understanding the mechanisms of metastasis in PCa patients and developing successful interventions.

**Methods:**

Using the GSE8511 and GSE27616 data sets, 21 metastasis-related genes were identified through the weighted gene co-expression network analysis (WGCNA) method. Subsequent functional analysis of these genes was conducted on the gene set cancer analysis (GSCA) website. Cluster analysis was utilized to explore the relationship between these genes, immune infiltration in PCa, and the efficacy of targeted drug IC50 scores. Machine learning algorithms were then employed to construct diagnostic and prognostic models, assessing their predictive accuracy. Additionally, multivariate COX regression analysis highlighted the significant role of POLD1 and examined its association with DNA methylation. Finally, molecular docking and immunohistochemistry experiments were carried out to assess the binding affinity of POLD1 to PCa drugs and its impact on PCa prognosis.

**Results:**

The study identified 21 metastasis-related genes using the WGCNA method, which were found to be associated with DNA damage, hormone AR activation, and inhibition of the RTK pathway. Cluster analysis confirmed a significant correlation between these genes and PCa metastasis, particularly in the context of immunotherapy and targeted therapy drugs. A diagnostic model combining multiple machine learning algorithms showed strong predictive capabilities for PCa diagnosis, while a transfer model using the LASSO algorithm also yielded promising results. POLD1 emerged as a key prognostic gene among the metastatic genes, showing associations with DNA methylation. Molecular docking experiments supported its high affinity with PCa-targeted drugs. Immunohistochemistry experiments further validated that increased POLD1 expression is linked to poor prognosis in PCa patients.

**Conclusions:**

The developed diagnostic and metastasis models provide substantial value for patients with prostate cancer. The discovery of POLD1 as a novel biomarker related to prostate cancer metastasis offers a promising avenue for enhancing treatment of prostate cancer metastasis.

## Introduction

Prostate cancer (PCa) ranks among the top ten most common malignant tumors in humans and is now the second leading cause of cancer-related mortality in men [1]. Despite advancements in diagnosis and treatment, the global incidence of PCa continues to increase annually. Treatment options for PCa include surgical resection, chemotherapy, radiotherapy, and hormonal therapy, all of which can effectively treat early-stage PCa characterized by localized tumors [2]. However, approximately 30% of PCa cases progress to metastatic disease, with bone metastases being the most prevalent in advanced cases [3]. The five-year survival rate for advanced metastatic PCa is only around 28% [4]. Since a significant portion of PCa cell growth relies on androgen receptor (AR) signaling, androgen deprivation therapy (ADT) remains the primary clinical treatment for advanced PCa [5]. While ADT typically induces remission for 1–2 years, the development of metastatic castration-resistant prostate cancer (mCRPC) leads to drug resistance and poor efficacy [6]. Therefore, there is a critical need to identify new therapeutic targets for metastatic PCa.

The tumor microenvironment (TME) is a key factor in promoting tumor progression and metastasis. Various elements like secreted molecules, changes in the extracellular matrix, vascular remodeling, and interactions with immune cells contribute to the dynamic nature of the TME. Tumor cells are responsible for orchestrating the intricate changes in the extracellular matrix, the formation of new blood vessels, and communication between cells within the TME [7]. Immune cells present in the TME, such as tumor-associated macrophages (TAMs) and infiltrating T cells, are known to drive the progression and spread of PCa. TAMs release pro-inflammatory cytokines such as CCL5 and CCL2, influencing the development and dissemination of PCa [8, 9]. CD8 + T cells play a role in anti-tumor immunity in PCa and are regulated by the N6-methyladenosine regulator YTHDF1 [10]. Mast cells are important cellular components in the TME and have been linked to poor outcomes in individuals with prostate cancer and other solid tumors. Recent research has highlighted mast cell-derived SAMD14 as a new regulator in the prostate tumor microenvironment [11]. Therefore, a comprehensive investigation into the relationship between PCa metastasis and immune cells is essential for uncovering the mechanisms underlying PCa metastasis.

Multi-omics analysis, utilizing artificial intelligence and machine learning techniques, aims to analyze multidimensional data to uncover hidden patterns and correlations related to diseases. These technologies play a crucial role in making clinically relevant decisions and applications [12–14]. Martelin et al. developed a machine learning model for cost-effective preliminary prostate cancer screening [15]. Altıntaş et al. also achieved high accuracy in predicting urethral stricture after TURP with their model [16]. Additionally, Yin et al. used machine learning to predict biochemical recurrence of prostate cancer [17]. This study utilized the WGCNA method on two PCa metastasis-related datasets (GSE8511 and GSE27616) to pinpoint genes associated with PCa metastasis. By integrating differential prognostic genes from the TCGA-PRAD dataset, 21 differential prognostic genes linked to metastasis were identified. Cluster analysis further validated the relationship between these genes and PCa patient prognosis, immunotherapy, and targeted therapy. Machine learning algorithms were employed to construct diagnostic and metastatic models to assess the predictive value of these genes for PCa diagnosis and metastasis. Multivariate COX regression analysis underscored the significance of POLD1 among metastasis-related genes. Moreover, significant associations between POLD1 and DNA methylation, as well as androgen receptor-related compounds, were observed. Immunohistochemistry was utilized to confirm the expression and prognostic variances of POLD1. In conclusion, this research identifies POLD1 as a novel marker of PCa metastasis, with implications for diagnosis, prognosis, metastasis, and immunotherapy.

## Materials and methods

### Datasets and patient samples

The study utilized the GSE8511 and GSE27616 datasets to investigate genes associated with prostate cancer (PCa) metastasis. GSE8511 included 13 metastatic PCa samples and 12 non-metastatic PCa samples, while GSE27616 had 4 metastatic PCa samples and 5 non-metastatic PCa samples. In addition, RNAseq data and clinical information from the TCGA-PRAD dataset were integrated into the analysis. The diagnostic model was developed and validated using the TCGA-PRAD, GSE6956, GSE16120, GSE14206, and GSE38241 datasets. Furthermore, the metastasis model was constructed and validated using the GSE8511, GSE32269, GSE27616, GSE29650, GSE41192, and GSE38241 datasets. Sixty PRAD tissue samples and corresponding adjacent tissue samples were obtained from Shanghai Outdo Biotech Company. The participants in the tissue chip study underwent surgical procedures between January 2011 and December 2014, with a follow-up period until November 2021, ranging from 6 to 10 years.

### WGCNA algorithm identifies metastasis-related genes

Utilizing the ‘WGCNA’ package, missing values in the GSE8511 and GSE27616 data are checked, and sample clustering is performed to identify outlier samples. Optimal power values were chosen to create a proximity matrix, ensuring that the gene distribution based on connectivity followed a scale-free network. Subsequently, topological overlap matrices are computed, gene clustering is conducted, dynamically spliced modules are identified, and similar modules are merged (each containing a minimum of 100 genes). Correlation coefficients and P values between different modules and clinical traits are calculated, assessing the relationship between modules and their constituent genes to identify those most closely associated with prostate cancer metastasis.

### Subgroup analysis based on metastatic genes

RNA-sequencing expression profiles and corresponding clinical information for PRAD were downloaded from the TCGA dataset. Consistency analysis was performed using the ConsensusClusterPlus R package (v1.54.0) with a maximum of 6 clusters and 80% of the total samples drawn 100 times. The clustering algorithm used was ‘hc’ with inner linkage method ‘ward. D2’. Clustering heatmaps were generated using the R software package pheatmap (v1.0.12).

### Immune infiltration and drug IC50 analysis

For a dependable assessment of the immune score findings, we utilized immunedeconv, a software package in R [18]. Each algorithm underwent comprehensive testing, and each presented distinct benefits. The XCELL approach was chosen for this research as it evaluates a broader spectrum of immune cells [19]. Chemotherapy response to various drugs for each sample in TCGA-PRAD was predicted using the Genomics of Drug Sensitivity in Cancer (GDSC) website. The prediction was carried out through the R package pRRophetic, which estimated the IC50 of the samples using ridge regression.

### Constructing diagnostic and metastasis models

In order to develop a PCa diagnosis model with high accuracy and stable performance, we integrated multiple machine learning algorithms into various algorithm combinations. These algorithms include elastic network (Enet), gradient boosting machines (GBM), glmBoost, the least absolute shrinkage and selection operator (Lasso), linear discriminant analysis (LDA), NaiveBayes, plsRglm, random forest (RF), Ridge, Stepglm, supported vector machine (SVM), and extreme gradient boosting (XGBoost). The training set comprised the TCGA-PRAD dataset, with validation performed on GSE6956, GSE16120, GSE14206, and GSE38241 datasets. Each algorithm combination was evaluated based on the AUC value, and the combination with the highest average AUC was selected as the optimal model. Metastasis models were constructed using the LASSO regression algorithm and assessed through 10-fold cross-validation. The metastasis model was trained on GSE8511 data set and validated on a combination of GSE29650, GSE27616, and GSE32269 datasets. The analysis was conducted using the R software glmnet package.

### Analysis of POLD1 correlation with DNA methylation and screening and docking of compounds

The correlation analysis between POLD1 expression and DNA methylation was conducted using the Shiny Methylation Analysis Resource Tool (SMART) website. SMART is an interactive web server specifically designed for DNA methylation analysis within the TCGA project [20]. The Connectivity Maps (CMaps) website was utilized to examine the correlation between POLD1 and androgen receptor-related compounds [21, 22]. Subsequently, the chosen compounds were subjected to molecular docking with POLD1 using the CB-Dock2 website. The vina score served as the parameter for evaluating molecular docking affinity, with lower scores indicating higher affinity between the receptor and the ligand [23]. Typically, a vina score below − 5 is deemed to have a more favorable impact.

### Analysis of POLD1 expression in prostate cancer tissue microarrays by immunohistochemistry

The tissue microarray was initially placed in an oven at 85 °C for 10 min, followed by soaking in xylene for 15 min and hydration using an ethanol concentration gradient of 100%, 95%, 80%, and 70%. Subsequently, the chip was treated with a citric acid solution in an autoclave to facilitate antigen retrieval. After cooling, the chips were rinsed with PBS and exposed to hydrogen peroxide for 20 min. Next, the POLD1 antibody (AF302114) was added and incubated at room temperature for 2 h. The tissue microarray was then rinsed three times with PBS and incubated with immunohistochemical secondary antibodies for 20 min at room temperature. Following three additional rinses with PBS, the microarray was stained with DAB and hematoxylin. Dehydration was carried out using an ethanol gradient of 70%, 80%, 90%, and 100%. Subsequently, immersion in xylene for eight minutes and blocking of the microarray took place. The immunostaining intensity score ranges from 0 to 3, where 0, 1, 2, and 3 correspond to no reaction, weak reaction, moderate reaction, and strong reaction, respectively. A scale is then applied based on the proportion of positive staining observed: scores of 1, 2, 3, and 4 represent ranges of 0–25%, 26–50%, 51–75%, and 76–100%, respectively. The final score is calculated by multiplying the strength score by the scale score. Interpretation of results is as follows: scores from 0 to 5 indicate low expression, while scores from 6 to 12 indicate high expression.

### Statistical analysis

POLD1 expression in PCa and normal tissues was determined by the Wilcoxon rank-sum test. Prognostic analysis was performed using the log-rank test. Statistical significance was defined as *p* < 0.05.

## Result

### Screening genes related to PCa metastasis

**Fig. 1:**
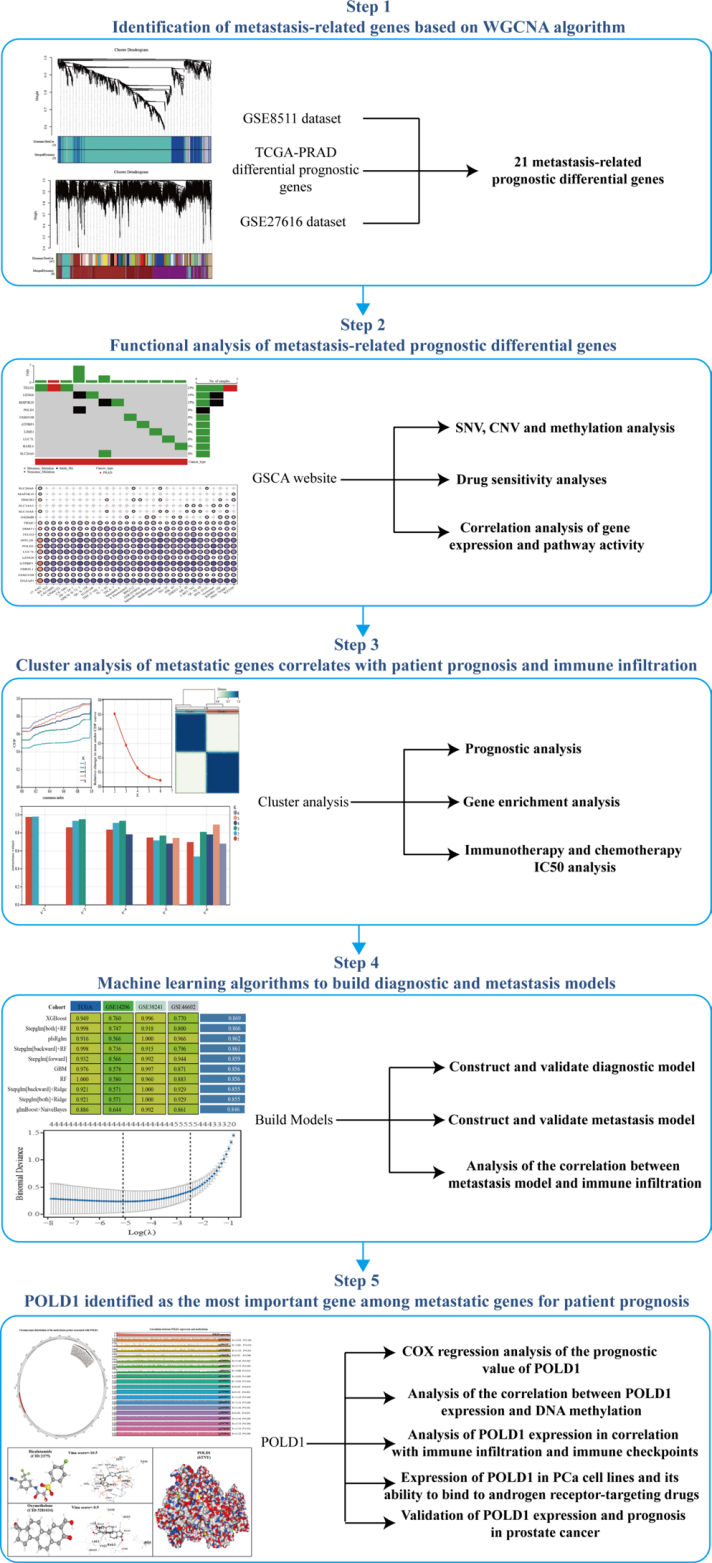
The workflow of our study

**Fig. 2:**
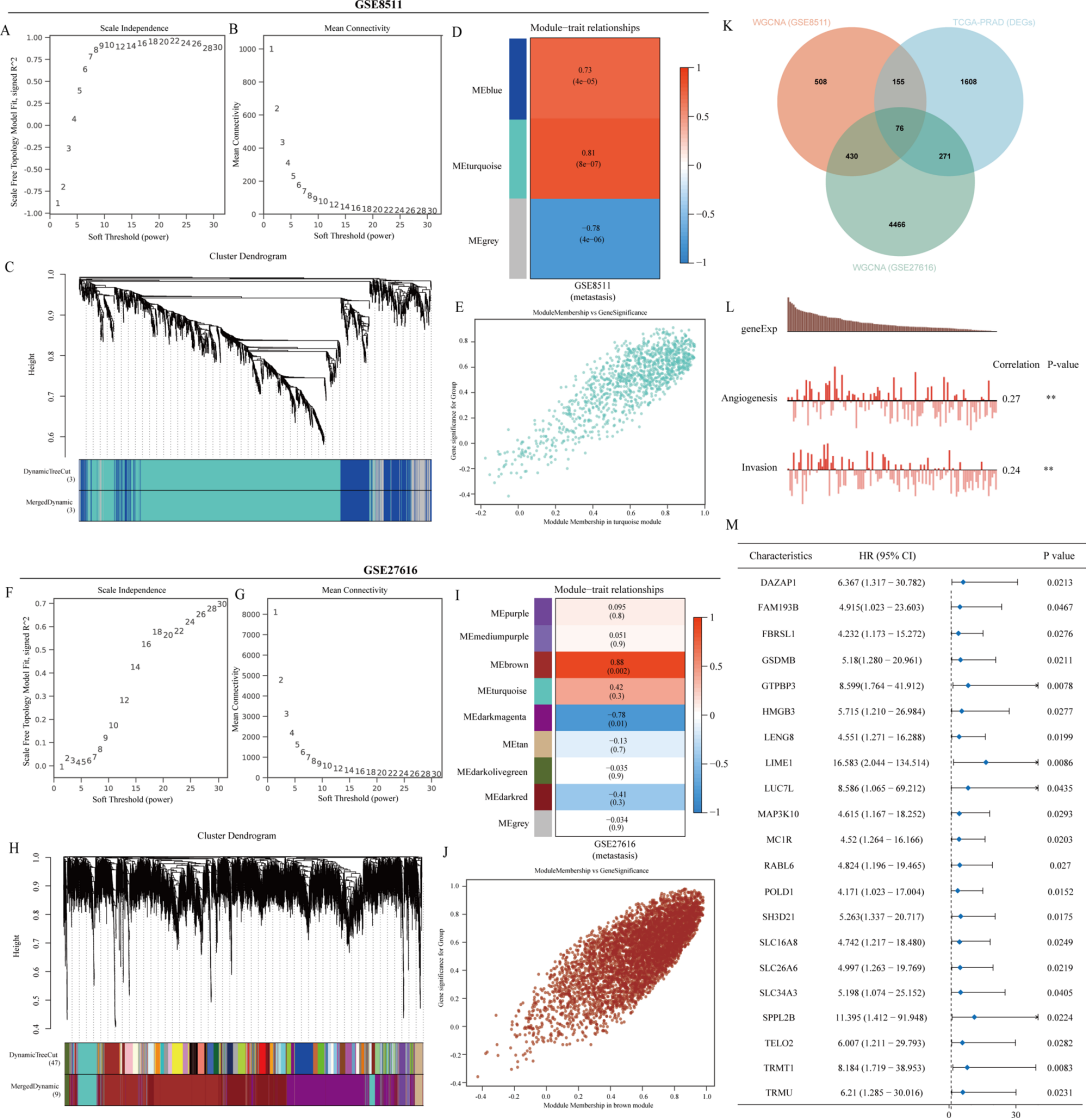
21 PCa metastasis-related prognostic genes identified. (**A-B**) The optimal soft-threshold power. The threshold was 8. (**C**) Weighted co-expression network modeling based on selected power values. (**D**) Heatmap of trait module associations. (**E**) Scatter plot of association between specific traits and module genes. (**F-G**) The optimal soft-threshold power. The threshold was 30. (**H**) Weighted co-expression network modeling based on selected power values. (**I**) Heatmap of trait module associations. (**J**) Scatter plot of association between specific traits and module genes. (**K**) Venn diagram plotting the intersection of GSE811 and GSE27616 metastases with the TCGA-PRAD oncogene. (**L**) Functional analysis of metastasis-related genes. (**M**) Prognostic analysis of metastasis-related genes

The workflow of our study was shown in Fig. 1. To identify potential targets for regulating PCa metastasis, we conducted WGCNA analysis on the GSE8511 dataset. To meet the requirements of scale-free network distribution, we determined the adjacency matrix weight parameter power value to be 8 (Fig. 2A-B). Subsequently, a weighted co-expression network model was constructed based on this power value, resulting in the categorization of all genes into 3 modules (Fig. 2C). By utilizing the Pearson correlation algorithm to assess the correlation coefficient and p-value between module eigengenes and traits, we observed that the turquoise module exhibited the highest correlation (correlation coefficient of 0.81) (Fig. 2D-E). Additionally, we incorporated GSE27616 into our analysis, where we selected a power value of 30 using the WGCNA method, leading to the division of genes into 9 modules based on this power value. Notably, we identified the brown module as having the strongest correlation (correlation coefficient of 0.88) (Fig. 2F-J). Regulating PCa metastasis is a critical aspect that drives cancer progression. To identify potential targets, we examined all genes up-regulated in cancer within the TCGA-PRAD dataset, resulting in the identification of 76 metastasis-related up-regulated genes (Fig. 2K). Leveraging ScRNA-seq technology, we delved into the functional heterogeneity of cancer cells. CancerSEA, a specialized database, was utilized to decode the distinct functional states of cancer cells at a single-cell level. By analyzing the functions of the 76 identified genes in the CancerSEA database, we discovered their association with PCa angiogenesis and invasion, validating their role in regulating PCa metastasis (Fig. 2L). Furthermore, we identified 21 genes among the 76 metastasis-related genes that were linked to poor prognosis in PRAD, highlighting their significance as risk factors (Fig. 2M).

### The role of metastasis-related genes in PCa

**Fig. 3:**
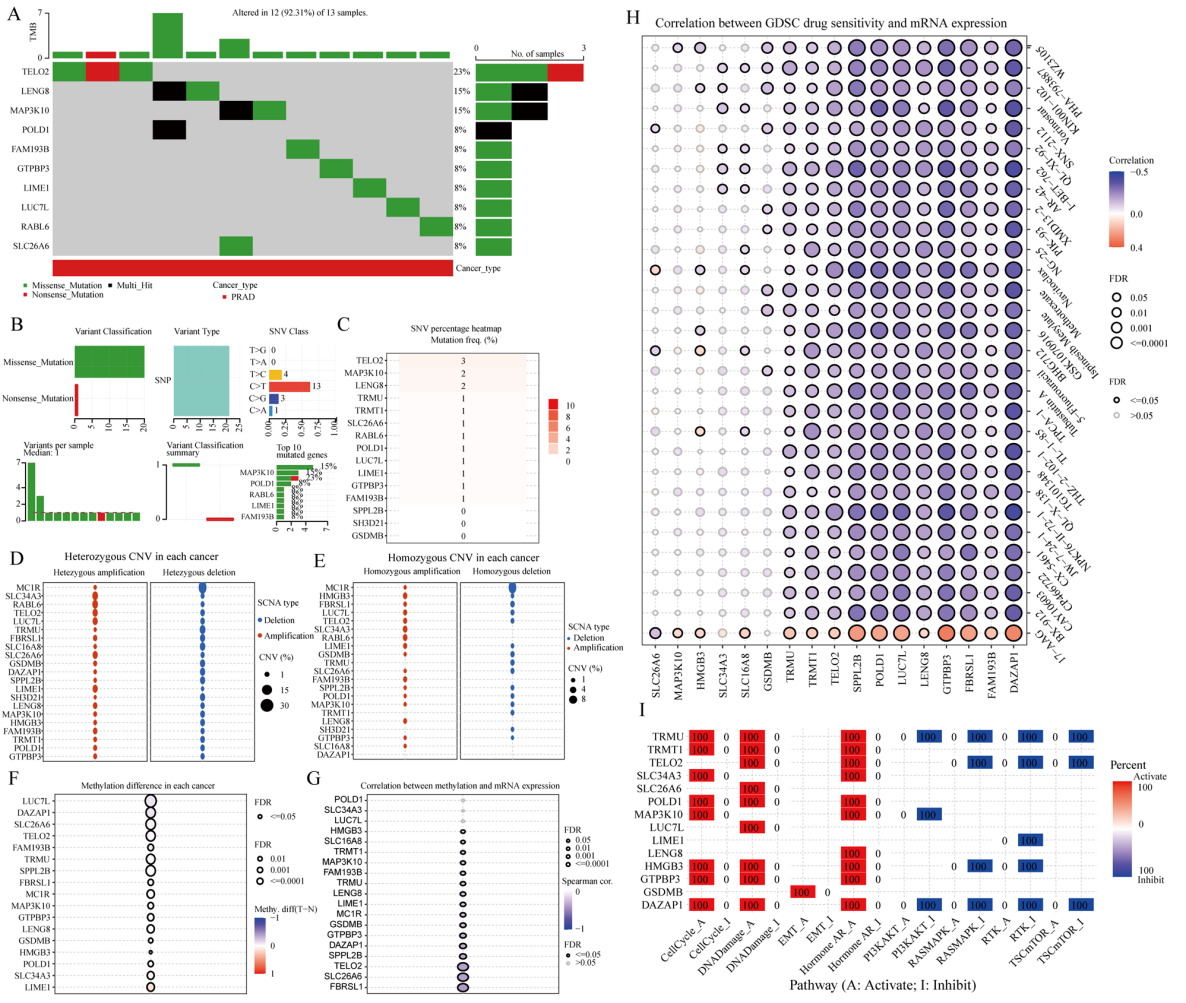
Metastasis-related prognostic genes play an important role in PRAD. (**A**) Oncoplot provides the situation of the SNV of the top 10 mutated genes. (**B**) Figure summarizes the SNV classes of inputted gene set. (**C**) Figure provides the profile of SNV. (**D**) Figure provides the profile of heterozygous CNV. (**E**) Figure provides the profile of homozygous CNV. (**F**) Figure summarizes the methylation difference between tumor and normal samples. (**G**) Figure summarizes the profile of correlations between methylation and mRNA expression. (**H**) Figure summarizes the correlation between gene expression and the sensitivity of GDSC drugs. (**I**) Figure summarizes the percentage of cancers in which specific gene’s mRNA expression has potential effect on pathway activity

The mutational status of genes in TCGA-PRAD was initially examined. An Oncoplot was used to visualize the SNV status of the top 10 mutated genes out of the 21 genes analyzed in PRAD. Among these genes, LENG8 and TELO2 exhibited the highest mutation frequencies (Fig. 3A). The SNV categories of the gene sets in PRAD were summarized, indicating a predominance of missense mutations (Fig. 3B). A heat map was created to depict the mutation frequencies of these genes, highlighting TELO2, LENG8, and MAP3K10 as the top 3 genes with the highest mutation rates (Fig. 3C). Furthermore, the analysis included the profiles of heterozygous and homozygous CNVs for the 21 genes in PRAD, with larger circles denoting higher frequencies (Fig. 3D-E). The study also investigated methylation variances between PRAD and normal samples, as well as the correlation between methylation and mRNA expression of these genes. Larger circles in the visualization indicated stronger correlations with methylation (Fig. 3F-G). Additionally, the association between these genes and chemotherapy drugs was explored using the GDSC database, revealing a high correlation between DAZAP1 and the chemotherapy drugs analyzed (Fig. 3H). Finally, the relationship between the expression of these genes and established pathways was assessed, showing that genes related to metastasis were closely linked to DNA damage and activation of androgen receptors (Fig. 3I). In conclusion, this comprehensive analysis provides insights into the potential roles of these genes in PRAD from various perspectives.

### Consistent clustering analysis based on metastasis-related genes

**Fig. 4:**
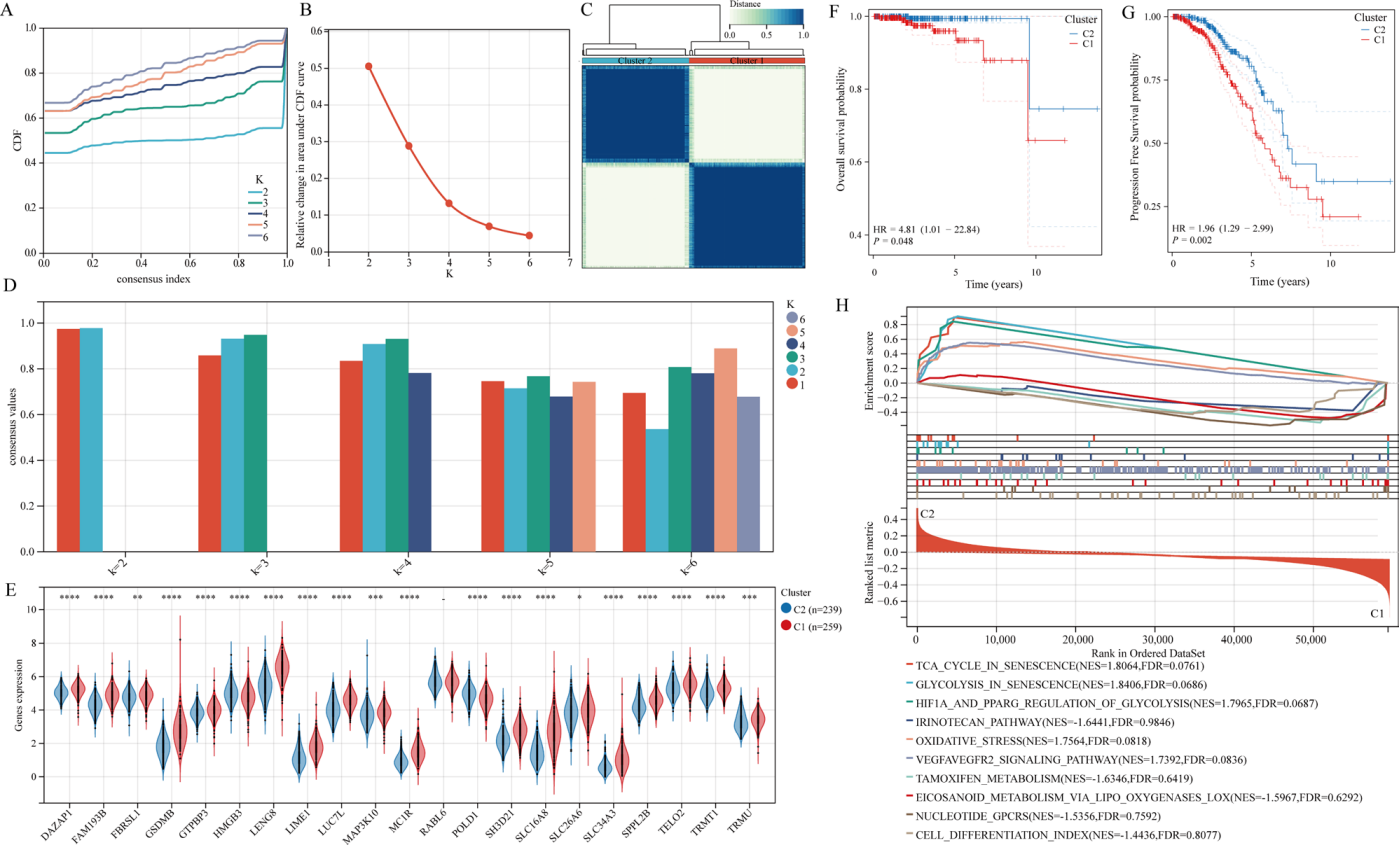
PRAD patients were categorized into two clusters based on the expression of metastasis-related genes. (**A**) Cumulative distribution curve. (**B**) Area under the distribution curve. (**C**) Clustering heatmap. (**D**) Sample clustering consistency. (**E**) Expression of metastasis-related genes in two clusters. (**F**) Difference in overall survival between the two clusters. (**G**) Difference in progression-free survival between the two clusters. (**H**) Gene enrichment analysis of two clusters

Consensus clustering analysis was conducted based on the expression of metastasis-related genes in the TCGA-PRAD data set. Determining the optimal number of clusters was achieved by identifying the K value with the lowest ‘Proportion of Ambiguous Clusters’ (PAC), a common approach in consensus clustering. PAC measures the intermediate part, defined as the consensus index within the range (u1, u2) ∈ [0, 1], where u1 is close to 0 and u2 is close to 1 (for instance u1 = 0.2 and u2 = 0.8). Lower PAC values indicate a smoother middle segment and fewer inconsistent assignments in permuted clustering runs [24]. The cumulative distribution curve and the area under the curve revealed that the highest average consistency within the groups was achieved when K = 2. Furthermore, a clustering heat map for K = 2 was generated (Fig. 4A-D). When K = 2, the TCGA-PRAD samples were divided into two clusters: cluster 1 with 259 samples and cluster 2 with 239 samples. Significant differences in metastasis-related genes, except for RABL6, were observed between these two clusters (Fig. 4E). When PRAD metastasizes, the patient’s prognosis often deteriorates. Consequently, we conducted an analysis to compare the overall survival and progression-free survival outcomes between two clusters. Our findings indicate that patients in cluster 1 consistently experienced a poorer prognosis in terms of both overall survival and progression-free survival (Fig. 4F-G). To investigate the underlying reasons and potential mechanisms contributing to the notable disparity in prognosis among the two patient clusters, we conducted GSEA. Our findings indicated that aging, oxidative stress, and other related factors could potentially have a significant impact on the outcomes (Fig. 4H).

### Correlation of metastasis-related genes with immunotherapy and targeted drug therapy for PRAD

**Fig. 5:**
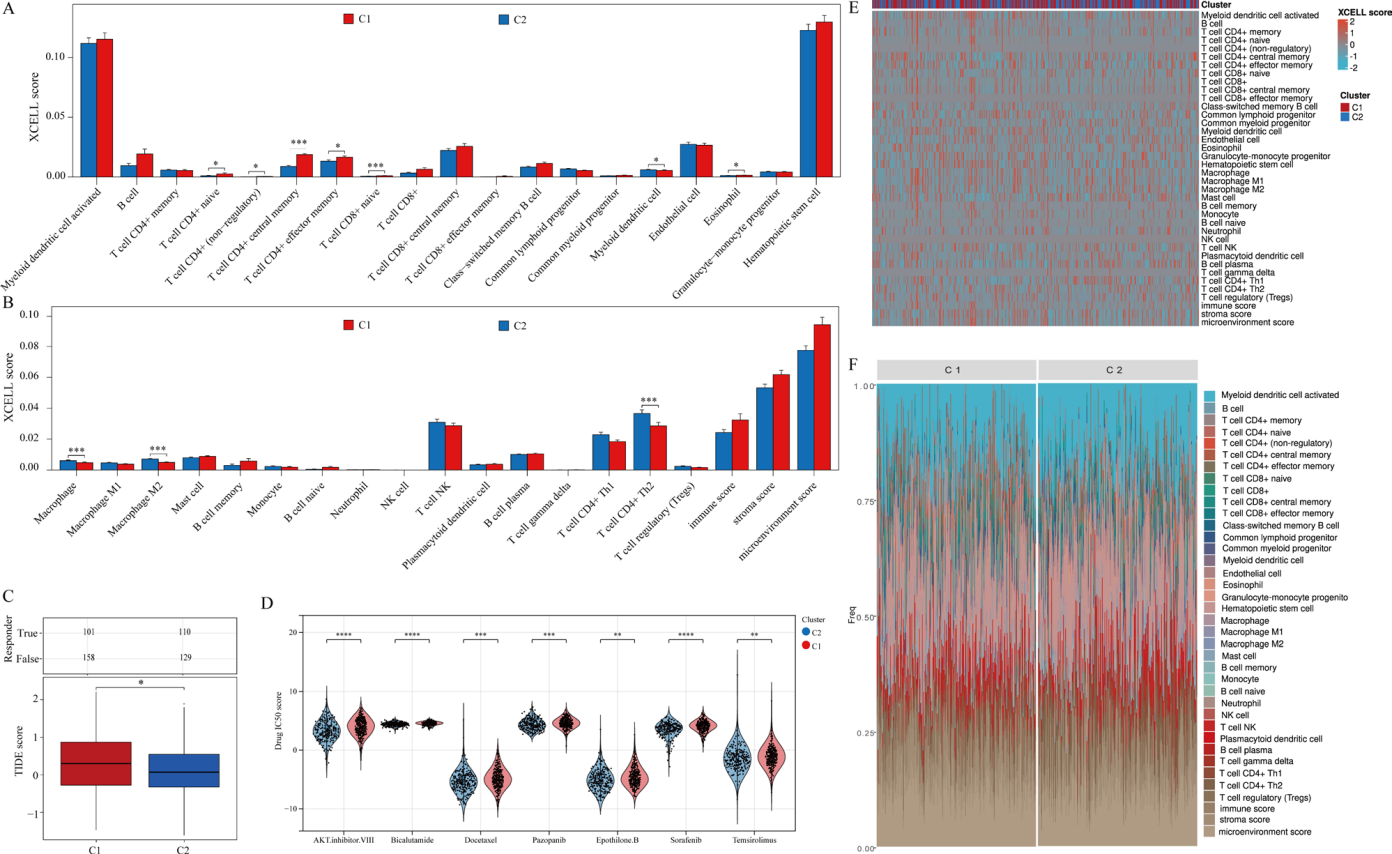
Metastasis-related genes are associated with PRAD immunotherapy and targeted therapy. (**A-B**) Analysis of the infiltration level of each immune cell in PRAD samples based on the XCELL algorithm. (**C**) Analysis of responsiveness of two clusters treated with immunosuppressive drugs. (**D**) Analyzing differences in IC50 scores for different targeted drugs between two clusters. (**E**) Heat map of different immune cell infiltration levels. (**F**) Percent abundance of tumor-infiltrating immune cells per sample

Utilizing the XCELL algorithm, our study delved into the relationship between metastasis-related genes and immune cell infiltration in PRAD. Within both clusters, notable variances were observed in T cell subsets (CD4 + naive, CD4 + non-regulatory, CD4 + central memory, CD4 + effector memory, CD8 + naive), myeloid dendritic cells, eosinophils, macrophages, and M2 phagocytes as opposed to CD4 + Th2 cells (Fig. 5A-B). Subsequent analysis of patient responsiveness to immunosuppressive treatment revealed a significantly higher tumor immune dysfunction and exclusion (TIDE) score in cluster 1 compared to cluster 2, potentially contributing to the less favorable prognosis in cluster 1 (Fig. 5C). Furthermore, examination of IC50 scores for commonly used clinical drugs between the clusters displayed significant differences across the seven targeted drugs assessed (Fig. 5D). Lastly, visualization of the immune cell infiltration levels in both clusters, along with the percentage abundance of tumor-infiltrating immune cells for each sample, was depicted through expression heatmaps generated using the XCELL algorithm (Fig. 5E-F).

### Build PRAD diagnostic models

**Fig. 6:**
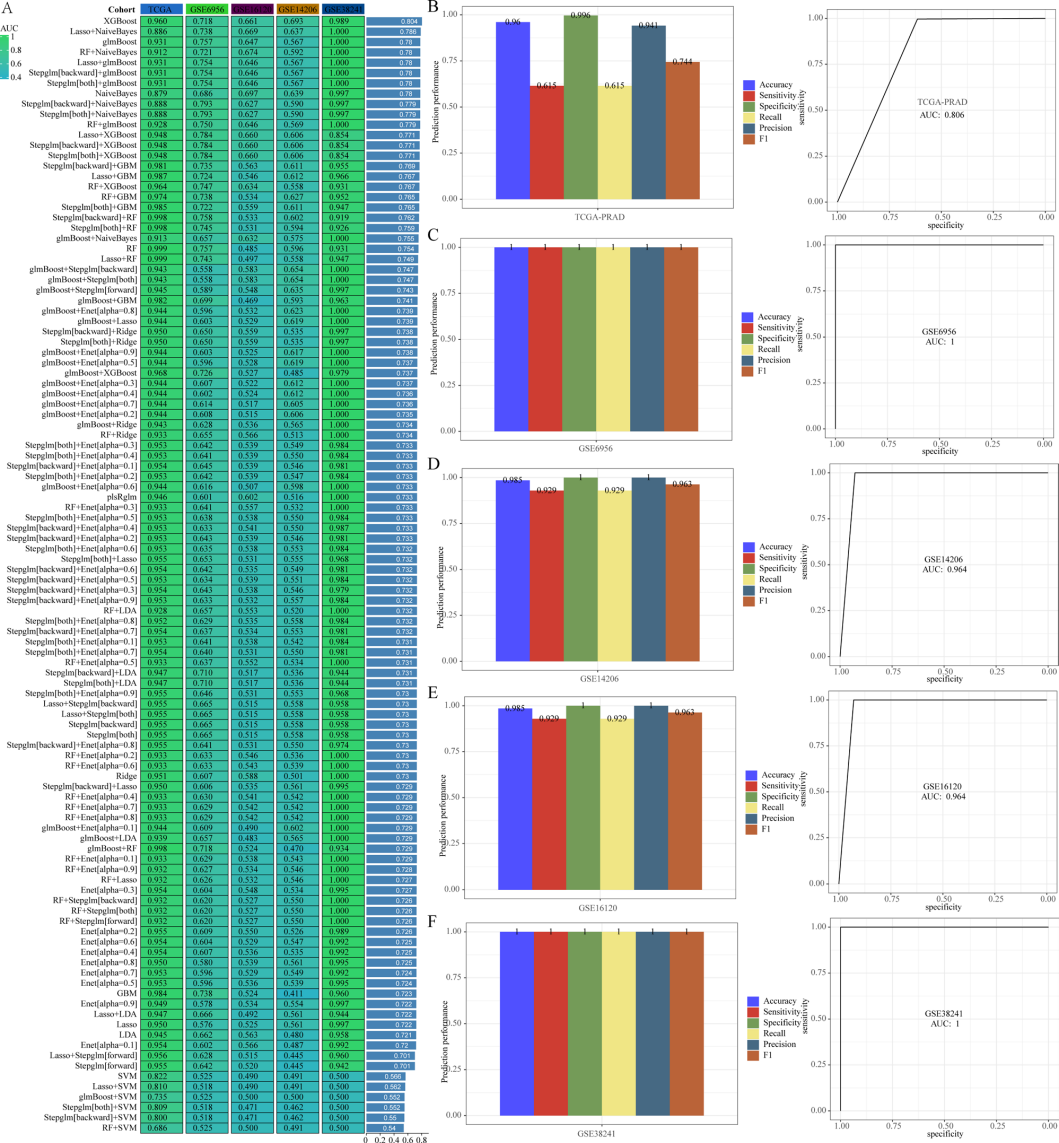
The model developed by the XGBoost algorithm is considered the top PRAD diagnostic model. (**A**) Comparison of AUC values among diagnostic models created by various algorithm combinations. (**B-F**) ROC curves for training and validation sets

To develop diagnostic models for PRAD, three datasets were utilized: TCGA-PRAD for training and GSE6956, GSE16120, GSE14206, and GSE38241 for validation. Among the 10 different machine learning algorithms tested, the XGBoost algorithm demonstrated superior efficiency in constructing diagnostic models (Fig. 6A). The AUC value for the TCGA-PRAD training set was 0.960, while the AUC values for the GSE6956, GSE16120, GSE14206, and GSE38241 validation sets were 0.718, 0.661, 0.693, and 0.989, respectively. The diagnostic model developed by the XGBoost algorithm identified 18 genes: DAZAP1, FAM193B, GSDMB, GTPBP3, HMGB3, LIME1, LUC7L, MAP3K10, MC1R, POLD1, RABL6, SH3D21, SLC16A8, SLC26A6, SPPL2B, TELO2, TRMT1, and TRMU. Based on these findings, the XGBoost algorithm was exclusively employed to construct diagnostic models for the aforementioned datasets, yielding consistently satisfactory results (Fig. 6B-F). Whether analyzing the TCGA-PRAD training set or the GSE6956, GSE16120, GSE14206, and GSE38241 validation sets, all AUC values exceeded 0.8, underscoring the exceptional predictive capability of our model.

### Build a metastasis prediction model

**Fig. 7:**
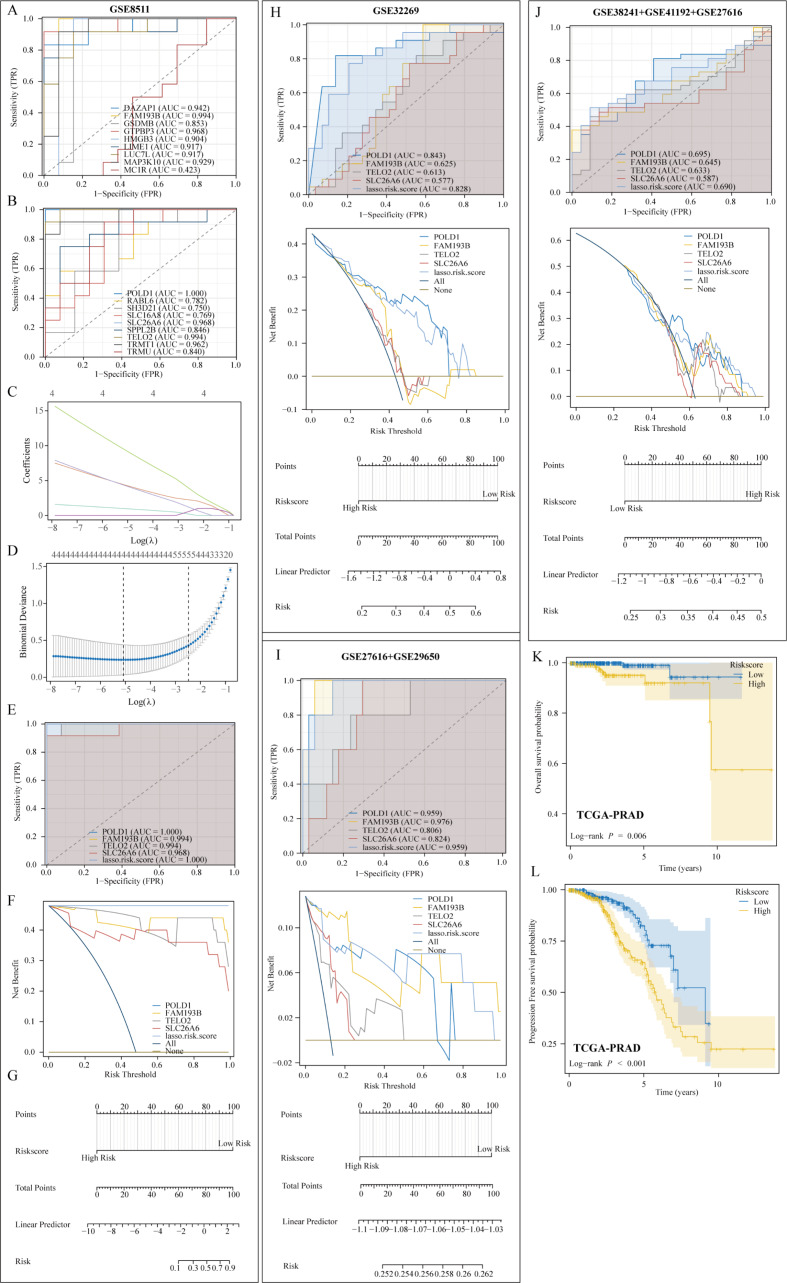
Construction of a metastasis prediction model based on metastasis-related genes. (**A-B**) ROC curves on metastasis genes in the GSE8511 dataset. (**C-D**) Construction of metastasis prediction model based on LASSO algorithm. (**E-G**) The metastasis prediction model has excellent predictive value. (**H-J**) Validating the predictive value of metastasis prediction models. (**K-L**) Correlation of high and low risk groups with overall and progression-free survival in patients with PRAD

This study focused on the significance of metastasis-related genes and utilized ROC curves to pinpoint the top 5 genes linked to metastasis in the GSE8511 dataset (Fig. 7A-B). Subsequently, a prediction model for PRAD metastasis was constructed based on the expression levels of these five genes employing the LASSO algorithm. The model incorporated 4 genes: FAM193B, POLD1, TELO2, and SLC26A6 (Fig. 7C-D). The formula for calculating the risk score is as follows: risk score = FAM193B * 0.98284 + POLD1 * 9.34847 + TELO2 * 4.34915 + SLC26A6 * 4.26241. Analysis of the ROC curve indicated that the model exhibited robust predictive performance (AUC = 1) for the metastasis model in PRAD patients (Fig. 7E). Further validation through decision curve and nomogram analysis confirmed the model’s predictive accuracy (Fig. 7F-G). Subsequent assessment in the GSE32269 dataset revealed an AUC value of 0.828, underscoring the predictive capability of the model (Fig. 7H). In order to overcome the limitation of the small sample size in GSE27616, the sample was amalgamated with other datasets for validation, consistently demonstrating the effectiveness of the metastasis model (Fig. 7I-J). Nonetheless, validation in the TCGA-PRAD dataset was not possible due to insufficient transfer samples. Nevertheless, an analysis of the correlation between risk score and patient prognosis in the TCGA-PRAD dataset revealed that patients classified as high-risk exhibited poorer overall and progression-free survival outcomes (Fig. 7K-L).

### Correlation analysis of metastasis model with PRAD immune infiltration and sensitivity to targeted drugs

**Fig. 8:**
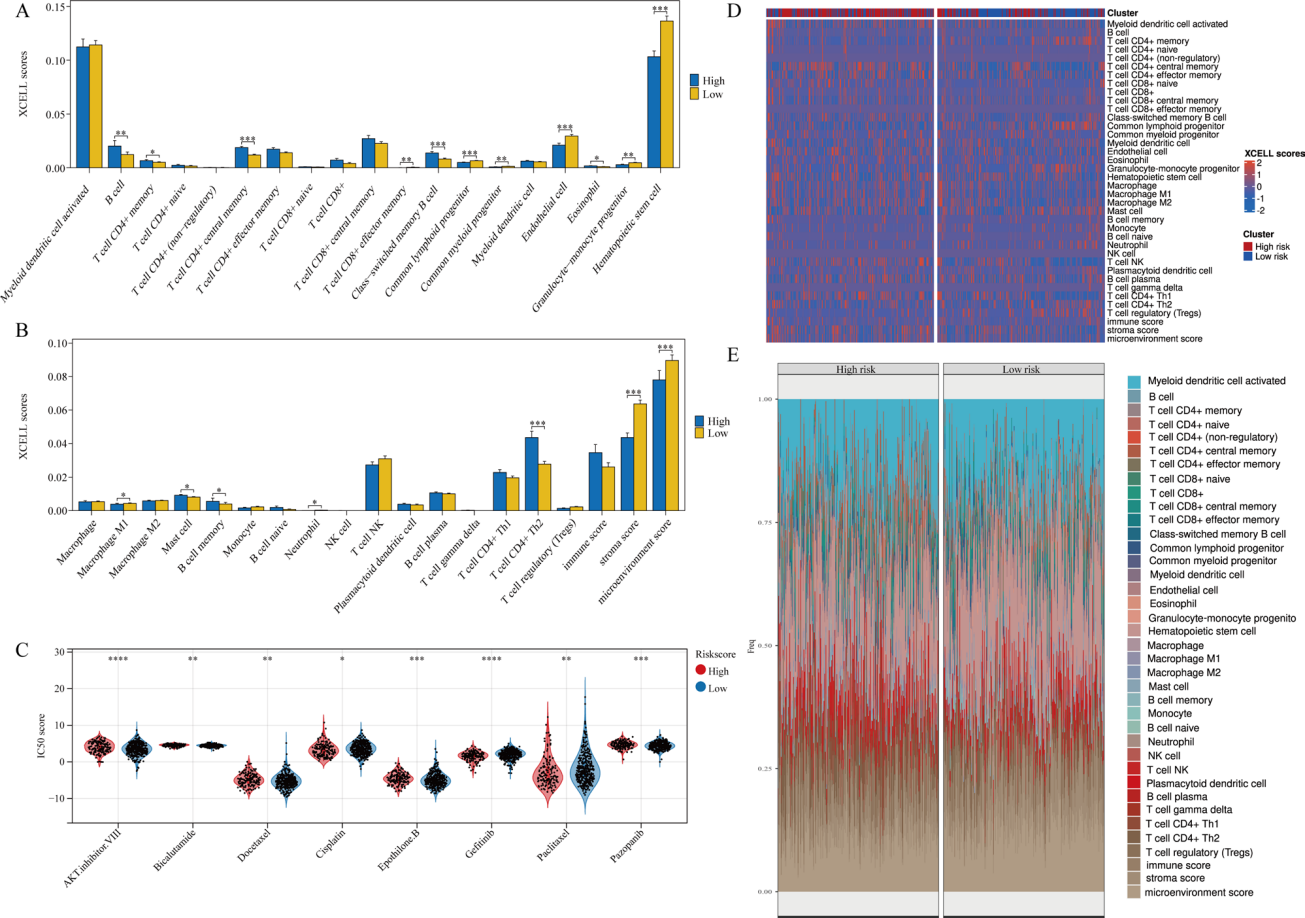
Metastasis model significantly correlates with PRAD immune infiltration and targeted drug sensitivity. (**A-B**) The degree of infiltration of each immune cell in the high and low risk groups was analyzed according to the XCELL algorithm. (**C**) Analyzing differences in IC50 scores for different targeted drugs between high and low risk groups. (**E**) Heat map of different immune cell infiltration levels. (**F**) Percent abundance of tumor-infiltrating immune cells per sample

We first explored the correlation between the metastasis model and PRAD immune cell infiltration, and we found that between high and low risk groups, B cell, T cell CD4 + memory, T cell CD4 + central memory, T cell CD8 + effector memory, Class-switched memory B cell, Common lymphoid progenitor, Endothelial cell, Granulocyte-monocyte progenitor, Mast cell, Neutrophil, B cell memory, T cell CD4 + Th2, stroma score and microenvironment score (Fig. 8A-B). Eight clinically commonly used targeted drugs were included for analysis, including drugs commonly used by prostate cancer patients like bicalutamide. Significant differences were observed between high and low risk groups for these 8 drugs (Fig. 8C). Additionally, the heat map analysis of immune cell infiltration levels and abundance between high and low risk groups, based on the XCELL algorithm, was also conducted (Fig. 8D-E).

### Identification of the best prognostic genes among metastasis-related genes

**Fig. 9:**
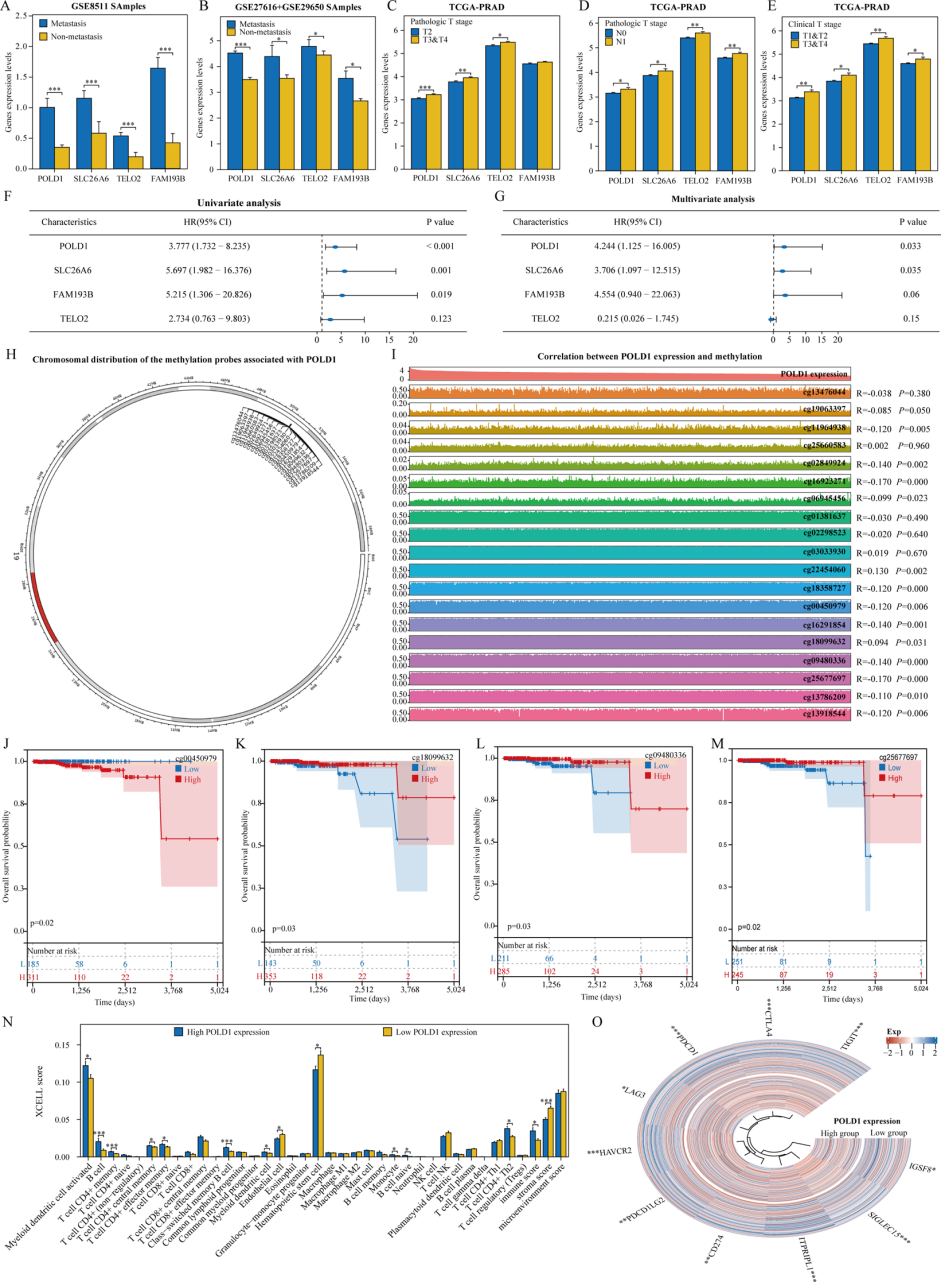
POLD1 identified as the best prognostic gene among metastasis-related genes. (**A**) POLD1 expression in metastatic and non-metastatic groups of the GSE8511 dataset. (**B**) POLD1 expression in metastatic and non-metastatic groups of the GSE27616 dataset. (**C**) Expression of POLD1 in pathologically different T-stages of the TCGA-PRAD dataset. (**D**) Expression of POLD1 in pathologically different N-stages of the TCGA-PRAD dataset. (**E**) Expression of POLD1 in clinically different T-stages of the TCGA-PRAD dataset. (**F-G**) Prognostic value of univariate and multivariate COX regression analysis of POLD1. (**H**) Chromosomal distribution of the methylation probes associated with POLD1. (**I**) Pearman correlation analysis between methylation probes and POLD1 expression. (**K-M**) Prognostic Kaplan-Meier (KM) curves for methylation probes. (**N-O**) Analysis of POLD1 correlation with immune infiltration and immune checkpoints

This study confirmed the significant roles of POLD1, SLC26A6, TELO2, and FAM193B in the diagnosis, metastasis, and prognosis of PRAD. To pinpoint metastasis-related genes crucial for prognosis, a thorough analysis was conducted. Examination of the GSE8511, GSE27616 and GSE29650 datasets revealed elevated expression levels of POLD1, SLC26A6, TELO2, and FAM193B in metastatic samples compared to non-metastatic samples (Fig. 9A-B). Correlation analysis in the TCGA-PRAD dataset indicated that POLD1, SLC26A6, and TELO2 expression was significantly higher in pathological T3 and T4 stages than in T2 stage, and in pathological N1 stage compared to N0 stage (Fig. 9C-D). Moreover, POLD1, SLC26A6, TELO2, and FAM193B expressions were notably lower in clinical T1 and T2 stages than in T3 and T4 stages (Fig. 9E). Subsequent univariate and multivariate COX regression analysis highlighted POLD1 and SLC26A6 as potential prognostic indicators for PRAD patients, with POLD1 standing out (Fig. 9F-G). Recent studies have demonstrated that abnormal DNA methylation of various genes, including metastasis suppressor genes and genes responsible for maintaining cell differentiation, plays a significant role in the progression of tumor metastasis [25]. By using the SMART website, we investigated the relationship between POLD1 and DNA methylation in PCa. In the TCGA-PRAD dataset, we identified a total of 19 methylation probes associated with POLD1(Fig. 9H). Out of these 19 probes, the expression of 13 methylation probes was found to be correlated with the expression of POLD1 (Fig. 9I). Subsequent prognostic analysis of these 13 POLD1-related methylation probes revealed that patients with high expression of cg00450979 had a poor prognosis, while patients with low expression of cg18099632, cg09480336, and cg25677697 had an even worse prognosis (Fig. 9J-M). Additionally, immune cell infiltration levels and correlations with immune checkpoint-related genes were compared between the POLD1 high expression group and the POLD1 low expression group (Fig. 9N-O).

### Analysis of POLD1 expression in PCa cell lines and its correlation with androgen-targeting compounds

**Fig. 10:**
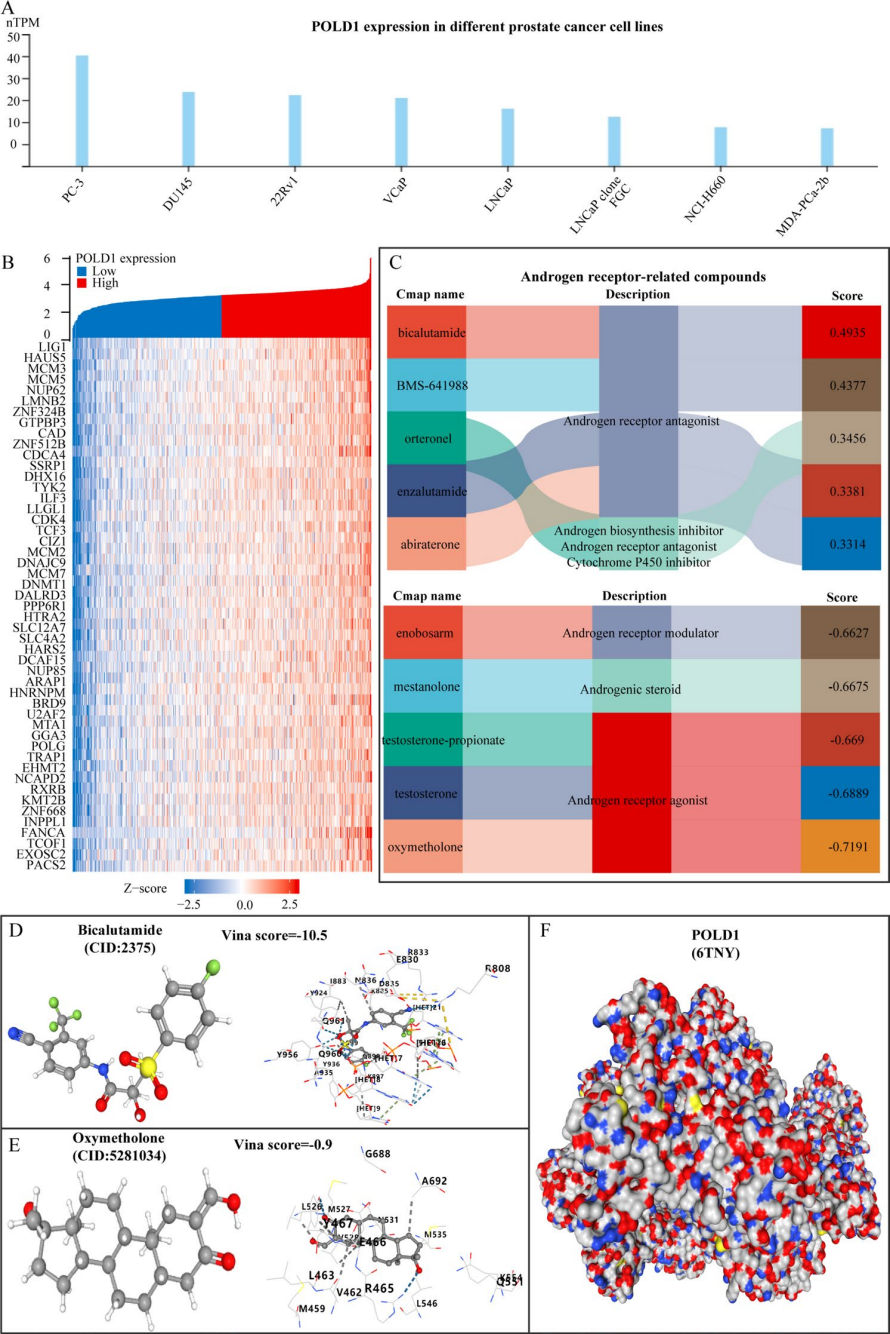
POLD1 has a high affinity for androgen receptor-targeting compounds. (**A**) POLD1 expression in PCa cell lines. (**B**) Heatmap of POLD1 co-expressed genes. (**C**) Androgen receptor-related compounds associated with POLD1. (**D**) Molecular docking of POLD1 with bicalutamide. (**E**) Molecular docking of POLD1 with oxymetholone. (**F**) Molecular structure of POLD1

Jin et al. integrated 45 The Human Protein Atlas (HPA) cancer cell lines with 973 The Cancer Cell Line Encyclopedia (CCLE) cancer cell lines, resulting in a total of 985 cancer cell lines for research purposes [26, 27]. The expression of POLD1 in prostate cancer cell lines was analyzed using tools provided by Jin et al., revealing that POLD1 was highly expressed in PC3 and DU145 cells, and least expressed in MDA-PCa-2b cells (Fig. 10A). Given that ADT is fundamental in treating metastatic prostate cancer, our objective was to identify compounds with the strongest correlation with POLD1 among androgen-related compounds. Initially, in the TCGA-PRAD dataset, we identified the top 50 genes positively correlated with POLD1 and generated a co-expression heat map (Fig. 10B). Utilizing CMaps to unveil mechanisms of action of small molecule drugs, functionally annotate genetic variations in disease genes, and guide clinical trials. We further analyzed compounds highly correlated with POLD1 in VCAP cells via the SMap website based on the top 50 positively correlated genes. From drugs targeting androgen receptors, we identified the top 5 drugs positively correlated with POLD1 and the top 5 drugs negatively correlated, which were then used for subsequent analysis (Fig. 10C). Subsequently, we presented the 3D structures of the significant positive correlation compound bicalutamide and negative correlation compound oxymetholone. Following this, molecular docking with POLD1 was conducted to confirm their binding affinities. Our analysis of molecular docking results showed promising outcomes, indicating that POLD1 exhibits a robust binding affinity towards both compounds (Fig. 10D-F).

### Expression and prognostic value of POLD1 in PRAD

**Fig. 11:**
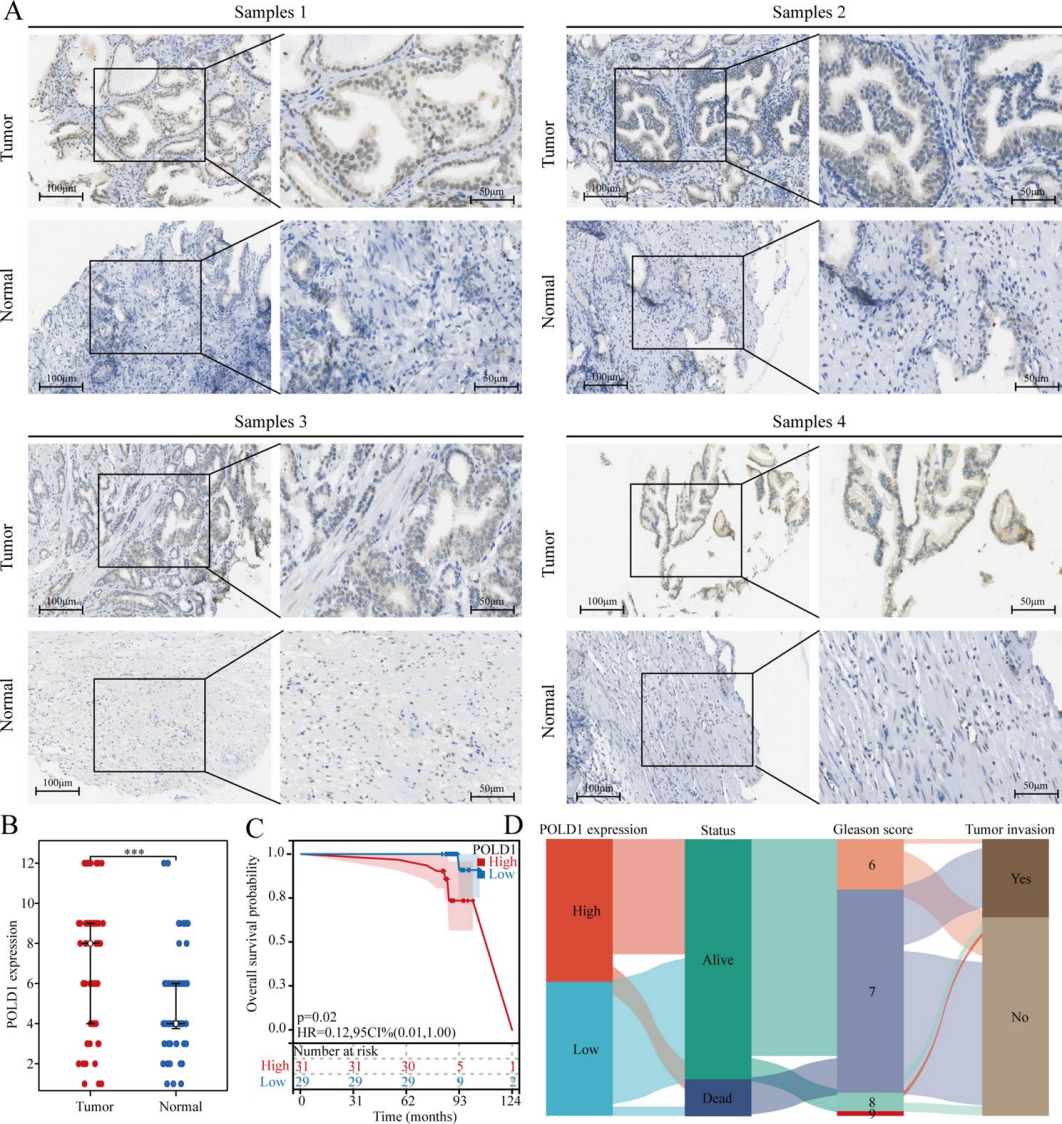
POLD1 is highly expressed in PCa and is associated with poor patient prognosis. (**A-B**) Differential expression of POLD1 in PCa. (**C**) KM curve of overall survival of POLD1 in PCa. (**D**) Sankey diagram showing distribution of patients in different subgroups

This study provides evidence supporting the significant role of POLD1 in prostate cancer. A total of 60 paired PCa samples and corresponding adjacent cancer samples were utilized for immunohistochemical staining to validate these findings. The main objective was to investigate the differences in POLD1 expression and its impact on the prognosis of PCa patients. The results demonstrated a notable increase in POLD1 expression in PRAD compared to normal prostate tissue (Fig. 11A). Dot plots were employed to visually depict the differences in POLD1 expression between PCa and normal tissue (Fig. 11B). Additionally, the analysis revealed a correlation between elevated POLD1 levels and poorer prognosis in PCa patients (Fig. 11C). Lastly, a Sankey plot was utilized to illustrate the distribution of patients with different characteristic variables based on POLD1 expression (Fig. 11D).

## Discussion

PCa mortality continues to be reduced by significant advancements in early detection and novel treatment modalities. Despite these progressions, PCa remains the most common malignancy among men globally [28]. Metastatic PCa accounts for around 400,000 fatalities annually. While the 5-year survival rate for localized PCa is nearly 100%, it drops to only 30% for metastatic PCa [29]. Thus, investigating markers associated with metastasis is crucial in identifying individuals at high risk of developing deadly metastatic tumors and offering improved treatment strategies for this patient population.

In our study, we initially identified PCa metastasis-related genes from the GSE8511 and GSE27616 datasets. By focusing on genes highly expressed in cancer that facilitate cancer progression, we cross-referenced these metastasis-related genes with the TCGA-PRAD dataset, resulting in 76 differential genes. Leveraging ScRNA-seq, we delved into the functional heterogeneity of cancer cells. CancerSEA, a specialized database, offers a comprehensive understanding of distinct functional states of cancer cells at a single-cell level [30]. Through the CancerSEA platform, we investigated the potential roles of these 76 genes in PCa. Our analysis revealed that metastasis genes primarily contribute to angiogenesis and invasion in PCa, supporting the significance of our findings as angiogenesis and invasion are critical factors in tumor metastasis. Previous studies have shown that tumor-derived exosomes and their contents play a role in promoting cancer metastasis. The interaction between exosome PGAM1 and ACTG1 can enhance PCa metastasis by regulating angiogenesis [31]. Furthermore, Ephrin-A2, a member of the Eph receptor subgroup, has demonstrated value in diagnosing and predicting the prognosis of PCa. It has also been found to facilitate PCa metastasis by promoting angiogenesis and EMT [32]. These findings collectively underscore the pivotal role of angiogenesis in PCa metastasis. Functional analysis revealed a significant correlation between metastasis-related genes and the activation of Hormone AR. It is widely acknowledged that the progression of PCa is influenced by androgens, and endocrine therapy targeting androgens is a crucial aspect of PCa treatment [33]. These conclusions once again confirmed the accuracy of our screened metastasis-related genes.

Clustering classification is a method used to group similar samples together based on commonalities and differences in the data, while separating dissimilar samples into distinct clusters to reveal unique characteristics and interactions. This approach is commonly applied in various fields such as disease diagnosis, prognosis analysis, gene therapy, epidemiology, and medical image analysis. In our study, we categorized PCa samples into two clusters using cluster analysis, with cluster 1 showing a significantly poorer prognosis compared to cluster 2. Gene enrichment analysis revealed enrichment of pathways related to cellular senescence, oxidative stress, and other biological processes. Cellular senescence is commonly recognized as a tumor-suppressing mechanism; however, senescent cells also exhibit heightened invasiveness and lymphangiogenic capabilities attributed to the development of a senescence-associated secretory phenotype [34]. Recent studies report that cellular senescence is associated with the spatial evolution of colorectal cancer toward a more metastatic phenotype [35]. Oxidative stress plays a crucial role in promoting various aggressive behaviors in tumors. Recent studies have shown that oxidative stress can lead to redox modifications of the protein kinase A β subunit, and in some cases, it can drive tumor metastasis by facilitating the RNF25-mediated degradation of ECAD protein in hepatocellular carcinoma [36]. Immune infiltration plays a crucial role in tumor progression. Utilizing the XCELL algorithm, we identified distinct scores of immune cells in two clusters. Patients with elevated TIDE scores exhibited reduced effectiveness of immune checkpoint blockade therapy and shorter survival post-ICB treatment [37]. Notably, cluster 1 displayed markedly higher TIDE scores compared to cluster 2, correlating with the unfavorable prognosis observed in patients within cluster 1. Machine learning methods offer a convenient approach for identifying characteristic genes, particularly in screening for metastasis-related genes in PCa diagnosis. The diagnostic model derived from these genes demonstrates excellent predictive capabilities. Furthermore, utilizing the LASSO algorithm to construct a metastasis model also yields strong predictive value for metastasis. This model also shows a significant association with immune infiltration in PCa. COX regression analyses and our findings analyzed by immunofluorescence experiments highlighted POLD1 as a key prognostic gene. DNA methylation is an epigenetic modification crucial for regulating individual growth, development, gene expression patterns, and genome stability without altering the DNA sequence. This modification is significant in development and can be consistently passed on during cell proliferation. Numerous recent studies have highlighted the close association between abnormal DNA methylation and the onset and progression of tumors and cellular transformation [38, 39]. Methylation analysis platforms like MethSurv and SMART offer a convenient way to explore the relationship between genes and DNA methylation [20, 40]. Through our research on the SMART website, we identified 4 methylation probes associated with POLD1. These probes not only show a connection to POLD1 but also demonstrate a correlation between their expression and the prognosis of patients with PCa. The CCLE and HPA websites offer researchers a convenient platform to investigate gene expression in tumor cell lines. Numerous studies have successfully utilized these databases for analysis and validation [41, 42]. Our own research identified variations in POLD1 expression among PCa cell lines using these resources. This discovery will be valuable for future cell experiments. Additionally, CMap is a valuable tool for identifying functional relationships between small molecule compounds, genes, and disease states [43]. Our investigation revealed that POLD1 exhibits the strongest correlation with bicalutamide and oxymetholone. Notably, bicalutamide is a commonly used targeted drug for clinical metastatic PCa, further supporting the potential of POLD1 as a drug target. Furthermore, our immunofluorescence analysis confirmed high expression of POLD1 in PCa, with patients exhibiting high expression levels showing a poor prognosis.

Our research is enhanced by the utilization of bioinformatics. Transcriptome data allows us to uncover gene expression patterns in various tissues, developmental stages, or environmental conditions, aiding in our comprehension of gene regulatory networks and biological processes’ regulatory mechanisms. Nonetheless, bioinformatics-driven research does come with limitations, as the quality of genomic data utilized directly impacts the accuracy and reliability of results. Therefore, we strive to incorporate multiple data sets in our studies to validate our findings, ensuring result accuracy. Nevertheless, additional experiments are required to further validate our conclusions.

## Conclusion

Various machine learning methods were employed in our study to pinpoint key genes linked to metastasis in PCa. Our research findings support the correlation between these genes and the treatment outcomes of patients with PCa, including responses to immunotherapy, chemotherapy, and overall prognosis. Notably, our study identifies POLD1 as a significant gene, with our experiments confirming its crucial role in predicting tumour invasion and prognosis in PCa patients. Furthermore, our investigation underscores the potential of targeting POLD1 for the development of novel drugs for PCa.

## Data Availability

The data that support the findings of this study are available from the corresponding author upon reasonable request.

## Abbreviations

PCa: Prostate cancer
WGCNA: Weighted gene co-expression network analysis
AR: Androgen receptor
ADT: Androgen deprivation therapy
mCRPC: Metastatic castration-resistant prostate cancer
TME: Tumor microenvironment
Enet: Elastic network
GBM: Gradient boosting machines
LASSO: The least absolute shrinkage and selection operator
LDA: Linear discriminant analysis
RF: Random forest
SVM: Supported vector machine
XGBoost: Extreme gradient boosting
TAMs: Tumor-associated macrophages
KM: Kaplan meier
HPA: The human protein atlas
CCLE: Cancer cell line encyclopedia
GDSC: Genomics of drug sensitivity in cancer
CMaps: Connectivity maps
TIDE: Tumor immune dysfunction and exclusion
